# To Ignore, to Join in, or to Intervene? Contextual and Individual Factors Influencing Cyber Bystanders’ Response to Cyberbullying Incidents

**DOI:** 10.3390/children13010113

**Published:** 2026-01-12

**Authors:** Nikolett Arató, Lilla Németh, Peter J. R. Macaulay

**Affiliations:** 1 Department of School and Educational Psychology, Psychology Institute, Eötvös Loránd University, 1064 Budapest, Hungary; 2 School of Science, University of Derby, Kedleston Road, Derby DE22 1GB, UK

**Keywords:** cyberbullying, empathy, moral disengagement, social desirability, online context, bystanders

## Abstract

**Background/Objectives:** Cyber bystanders can choose from several different strategies during cyberbullying incidents and have a significant effect on the situation. Hence, cyber bystanders are specifically targeted by prevention programmes and research investigating variables influencing cyber bystander responses is crucial for such programmes. The aim of our study was (1) to explore contextual factors’ effect on cyberbullying incidents’ perceived severity and (2) the most frequent cyber bystander responses. We also aimed (3) to learn how the context of cyberbullying incidents affects cyber bystander responses and the joint effect of individual and contextual variables on cyber bystander responses. **Methods:** In total, 314 Hungarian high school students participated in our online survey (mean age = 16.15, SD = 3.28). The respondents filled in self-administered questionnaires that measured cyber bystander responses, severity of different cyberbullying incidents, empathy, moral disengagement, social desirability, and cyberbullying engagement. **Results:** First, our results showed that the respondents perceived public and visual cyberbullying, and when the victim was upset by it the most severe incidents. Second, in almost every condition, the two most likely cyber bystander responses were ignorance and emotional support for the victim. Third, the individual and contextual variables had a joint effect influencing cyber bystander responses except for emotional support to the victim that was only influenced by individual variables, i.e., empathy, moral disengagement, and social desirability. **Conclusions:** All in all, our results showed that all cyberbullying contexts were associated with cyber bystander responses and the prominent association between moral disengagement, social desirability, empathy, and prosocial cyber bystander responses. Moreover, these results could guide cyberbullying prevention to focus on cyber bystanders’ empathy training, decreasing their moral disengagement, and educating them about the effects of online contextual variables.

## 1. Introduction

The Internet offers various opportunities for adolescents; however, some online features can be used as means for disruptive behaviours such as hacking, cyberstalking, and cyberbullying [1]. As cyberbullying is often identified as a subtype of traditional bullying [2,3], researchers use the definitional criteria of traditional bullying (i.e., intention to cause harm, repetition of the behaviour, and an imbalance of power between the victim and perpetrator [4]) to inform our understanding of cyberbullying. On the other hand, cyberbullying is also characterised by unique features associated with the online domain, such as anonymity, publicity, and its 24/7 nature [5,6,7,8]. Due to these unique characteristics, cyberbullying can occur in the presence of a large audience [9,10], resulting in unrestricted dissemination and long accessibility of the harmful content [10]. The perpetrator’s dominance lies with their potential anonymity and their sophisticated use of digital technologies and online communications [11,12]. According to Zhu and colleagues [13], the prevalence of cybervictimisation ranged up to 57.5%, whereas the prevalence of cyberbullying perpetration ranged up to 46.3%. This highlights the widespread engagement in cyberbullying that is concerning given how cyberbullying can contribute to severe psychological, physical, social, and behavioural consequences [14]. Hence, prevention is increasingly crucial to protect adolescents from the adverse effects of cyberbullying engagement, and one key ‘element’ of prevention programmes that have been implicated in the literature is the role of those who witness cyberbullying, cyber bystanders [15].

### 1.1. Cyberbullying and Cyber Bystanders

Cyber bystanders can take on one of three roles during a cyberbullying incident: they can choose to reinforce the perpetrator, remain passive by ignoring the incident, or intervene to help the victim [16,17]. The cyber bystander responses can either be constructive or aggressive. For example, constructive interventions can be targeted toward the victim (e.g., seeking help from a trusted adult, providing emotional support to the victim, etc.), or to the bully (e.g., asking the bully to stop, encouraging them to apologise, etc.). Aggressive intervention means aggressive responses to the perpetrator (e.g., threatening them or humiliating them by posting embarrassing photos/videos of them online: 17). These cyber bystander roles are fluid, as cyber bystanders can take multiple different roles during the same, or different, cyberbullying incidents [18]. This fluidity of cyber bystander roles might be the consequence of the specific features of the online context [17]. From a theoretical perspective, the social psychological work of Latané and Darley [19,20] provides some explanation on cyber bystanders’ responses to cyberbullying. Their model outlined five steps of bystander intervention: (1) noticing the incident, (2) recognising the need for assistance, (3) feeling personal responsibility, (4) assessing the ability to help, and (5) deciding to help. However, these steps may be modified by the unique features associated with the online domain, e.g., online distractions, online multitasking [21,22], and situational ambiguity (i.e., the victim’s response is not immediately visible, or it is censored [23,24,25,26,27]). Most importantly, the potential large virtual audience witnessing the incident might result in cyber bystanders feeling less responsible to intervene [28,29] and/or they might feel others have a greater responsibility to intervene (e.g., friends of the victim) [18,30,31,32]. Apart from the previously mentioned determinants, there are two main types of factors implicated in the literature that influence cyber bystander responses: contextual and individual factors [33].

### 1.2. Contextual Factors and Cyber Bystander Responses to Cyberbullying

There is an array of contextual factors associated with cyber bystander responses to cyberbullying and a systemic literature review [33] organised these factors into ten main categories. These were (a) friendship with the perpetrator or victim; (b) social environment; (c) bystander effect [19,20]; (d) incident severity; (e) action of other bystanders; (f) request for assistance from the victim; (g) evaluation of the situation; (h) knowledge of effective strategies to address cyberbullying; (i) characteristics of virtual environments [34]; and (j) fear of retaliation.

Based on the results of Macaulay and colleagues [35], the current research investigated the perceived *severity* of the situation, the *publicity* of the incident, the degree of *anonymity*, the *type* of the cyberbullying, and the *victim’s response* to the situation as possible influential contextual factors in cyber bystanders’ responses. When cyber bystanders perceive the cyberbullying incident as severe, they are more likely to support and help the victim [18,24,30,36,37]. Regarding the severity of cyberbullying acts, young people found public and anonymous acts of cyberbullying more severe than something that occurred privately or when the perpetrator’s identity was known [35,38,39]. In addition, recent research has identified that cyber bystanders perceived visual types of cyberbullying (i.e., manipulation or embarrassing photos) as more severe compared to written acts of cyberbullying (i.e., an aggressive intentional text message), and perceived severity was further heighted when the victim was upset [35]. However, the application of Latané and Darley’s [19,20] bystander effect model can be more challenging to apply to the online domain. For instance, while the physical presence of other bystanders would inhibit bystander intervention via diffusion of responsibility, the potential greater number of virtual onlookers to an incident of cyberbullying may also reframe cyber bystanders intervening to help the victim [40]. Indeed, some studies have reported evidence of the notion that with more cyber bystanders present (i.e., a larger element of publicity), cyber bystanders are less likely to intervene [41,42]. However, this association is not linear between the number of cyber bystanders and prosocial behaviour [41].

So far, most of the research investigating anonymity in relation with cyber bystander responses has been focused on how cyber bystanders’ anonymity influences their behaviour during cyberbullying incidents [43,44]. Macaulay and colleagues [35], in a sample of 990 11–20-year-olds in England, suggested that when the cyberbullying was anonymous, cyber bystanders were more likely to ask for help from an adult or a friend to emotionally support the victim and to intervene and challenge the cyberbully. Regarding the type of cyberbullying, although its effect on perceived severity was studied before [8,45,46,47,48], only one study [35] investigated its effect on cyber bystander responses. According to the results of this study, young people were more likely to ask for help from a friend when the form of cyberbullying was written verbal than when it was visual [35]. Furthermore, the victim’s reaction is also crucial for cyber bystanders’ decisions about how to respond [21], as they are more likely to support, comfort, and help the victim when they express their negative feelings following their cybervictimisation [33,35,40]. Specifically, cyber bystanders were more likely to ask for help from an adult or from a friend, emotionally support the victim, and to intervene and challenge the cyberbully when the victim was upset as an outcome of the cyberbullying incident.

The facilitating effect of contextual variables in mobilising cyber bystanders seems prominent, however, there are still only a few studies targeting the deeper understanding of these variables [33]. For example, the effect of anonymity and the type of cyberbullying in mobilising cyber bystanders is supported by only one study’s results [35], hence there is need for further evidence regarding these contextual factors’ effects. Apart from the low number of studies, the inconsistences in the results also emphasise the need for further research. For example, there are contradictory results regarding the effect of publicity on cyber bystanders’ responses, as some results (e.g., [35]) showed that greater publicity facilitates cyber bystander intervention, whereas other studies (e.g., [41,42]) showed that the higher number of witnesses inhibits cyber bystander mobilisation. Consequently, there is a lack of consensus about the role of contextual factors in cyber bystander responses supported by only a few studies, so there is a significant need for the further investigation.

### 1.3. Individual Factors and Cyber Bystander Responses to Cyberbullying

In addition to contextual factors, individual factors also explain cyber bystander intervention to cyberbullying. The aforementioned systemic review [33] also identified several individual factors that affect cyber bystander responses. These were (1) empathy, (2) moral disengagement, (3) behavioural determinants, such as attitudes toward cyberbullying, self-efficacy beliefs, self-esteem, impulsivity, social anxiety, etc., (4) previous experience in bullying and cyberbullying, (5) demographic and socio-economic characteristics, and (6) social environment that includes cultural and peer group norms and social support from family and friends, among other factors. Based on the results of previous systemic analyses [21,33], the current study investigated the role of empathy, moral disengagement, social desirability, and previous cyberbullying experiences as the individual factors in influencing cyber bystanders’ responses besides the contextual variables.

Empathy is a socio-emotional competence that has a prominent effect on prosocial behaviour [49,50,51]. In the context of cyber bystander responses to cyberbullying, empathy enhances constructive intervention, as adolescents showing higher levels of empathic reasoning were more likely to intervene as a cyber bystander by helping, supporting, and comforting the victim [29,32,41]. While empathy has a key role on constructive cyber bystander responses, research on the subtypes of empathy (i.e., affective empathy and cognitive empathy) showed mixed findings. Schultze-Krumbholz and colleagues [52] showed that both affective and cognitive empathy increased the likelihood of prosocial cyber bystander responses and decreased the likelihood of reinforcing the perpetrator. However, the role of cognitive empathy is quite unclear as three different paths regarding its role emerged: (a) cognitive empathy having no influence on cyber bystander responses [53], (b) cognitive empathy having the sole effect [54], (c) cognitive empathy inhibiting cyber bystander intervention [55]. These discrepancies in findings regarding the role on different types of empathy highlight the need to further explore this individual factor in the current study.

Moral disengagement—a cognitive mechanism consisting of eight strategies that enables individuals to excuse or justify their actions by using this cognitive strategy [56]—is a further individual factor that has been implicated in the literature. Previous research [18,29,30] showed that cyber bystanders use moral disengagement strategies to justify their actions in cyberbullying incidents. For instance, cyber bystanders attribute blame to the victim to justify why they reinforced the perpetrator of cyberbullying, or why they remained passive during the cyberbullying incident [26,30]. Further mechanisms to justify passive cyber bystander response are attributing the responsibility to help to others [18,30,31,32] and downplaying the severity of the cyberbullying incident and the damage caused to the victim [24,28]. Moral disengagement can also be a determinant of aggressive cyber bystander response as adolescents with more likeliness to use moral disengagement were more likely to threaten the cyberbully and spread rumours about them [17,57]. On the contrary, cyber bystanders who do not use moral disengagement strategies take responsibility in intervening [58] and they are more likely to help, support, and/or defend the victim [18,24,30,37,58].

The peer group and its social norms can also influence cyber bystanders as there are certain behavioural rules that the group members are expected to follow [59]. For example, if ignorance was the accepted reaction to peer aggression in the peer community, cyber bystanders were more likely to remain passive onlookers in cyberbullying incidents [60]. Similarly, if there was a positive attitude towards cyberbullying behaviour in the peer group, youngsters were more likely to join the perpetrator [33,59,61], and if prosocial behaviour was highly appreciated in the peer group then adolescents were likely to support the victim [18,32,33,42,62]. Moreover, if adolescents perceived that there will be negative consequences in their offline social environment if they support the victim they did not intervene [21]. As such, this suggests the prominent role of conforming one’s behaviour to the group’s expectations through social desirability [33].

Furthermore, previous cyberbullying engagement has been implicated as an individual factor that may contribute to cyber bystander responses to cyberbullying. The findings showed that young people who previously experienced cybervictimisation were more likely to support the victims [29,63], whereas those who previously had perpetrated cyberbullying were more likely to show negative cyber bystander responses such as forwarding the harmful message and disseminating it to a broader audience [64].

The aforementioned research highlights that individual factors are prominent to understand cyber bystander responses. However, the role of empathy’s subtypes is still unclear, thus it needs further investigation and the number of studies focusing on the role of cyber bystanders is still limited [33]. Meanwhile, the already existing research includes a limited array of variables, so our research aimed to add more variables (social desirability) to the existing framework and already studied ones (empathy, moral disengagement), as there is a need for further attention considering the role of cyber bystanders [65].

### 1.4. The Current Study

The current study aimed to investigate the role of contextual [publicity, anonymity, type of cyberbullying, and victim’s response] and individual [empathy, moral disengagement, social desirability, cyberbullying engagement] variables on cyber bystanders’ responses [ignorance, reinforcement of the perpetrator, asking for help from an adult or a friend, emotional support for the victim, and direct intervention against the perpetrator]. First, the study aimed to explore the perceived severity of cyberbullying and cyber bystander responses with the following hypotheses:

**H1:**  *Perceived severity would be higher in the public domain, when the perpetrator is anonymous, the type of cyberbullying is visual, and the victim is upset.*

**H2:**  *Perceived severity would influence cyber bystander responses, i.e., cyberbullying contexts perceived as severe would elevate constructive cyber bystander responses.*

Second, the study also aimed to explore which cyber bystander response is the most frequent and to investigate how the manipulated context influenced cyber bystander responses, with the following hypotheses:

**H3:**  *Constructive and prosocial cyber bystander responses* *would be more likely to occur in the public domain, when the perpetrator is anonymous, the type of cyberbullying is visual, and the victim is upset. Meanwhile, non-constructive bystander responses would be more likely to occur in the private domain,* *when* *the perpetrator is not anonymous, the type of cyberbullying is written, and the victim is not upset.*

Third, the research aimed to explore the individual variables together with the contextual influence on cyber bystander responses:

**H4:**  *Higher levels of empathy, social desirability, and previous cybervictimisation would be associated with constructive cyber bystander responses in public, anonymous, and visual cyberbullying when the victim is upset. In contrast, we presumed that moral disengagement and previous cyberbullying perpetration would be associated with cyber bystander responses such as ignorance and reinforcing the perpetrator in private, not anonymous, and written verbal cyberbullying when the victim was not upset.*

## 2. Materials and Methods

### 2.1. Participants

Altogether, 320 high school students participated in the study, but six students’ data were removed due to incomplete answers and withdrawal from the participation. Thus, in the final sample there were 314 high school students (31.5% female) aged between 14 and 20 years (*M* = 16.15, *SD* = 3.28). The sample comprised 99 females (31.5%), 185 males (58.9%), 3 non-binary individuals (1.0%), and 1 person (0.3%) did not disclose their gender. The sample was collected from two types of schools: 65.9% of the respondents attended high schools and 33.8% of the students attended technical schools. Regarding the mothers’ educational level, 30% of the students’ mothers had university degree, and 20–20–20% had a college degree, high school, or technical school degree. Regarding the fathers’ educational level, 23% of the students’ fathers had university degree, 19% had college degrees, and 27% had high school degrees. In the case of both mothers and fathers, about 2–2% had elementary school degrees or lower education. To see the frequency of cyber bystander, cyberbullying perpetration, and cybervictimisation in our sample, see Supplementary Table S1.

### 2.2. Measures

Hypothetical cyberbullying vignettes [35] were used to measure cyber bystander responses and to assess the perceived severity of cyberbullying incidents. Four factors were manipulated in the hypothetical cyberbullying scenarios: publicity [public/semi-public/private], anonymity [anonymous/not anonymous], type of cyberbullying [written verbal/visual], and the victim’s response [upset/not upset]. The scenarios were presented in a random order to the participants, who were asked after each scenario to answer two questions. The first question addressed the perceived severity of the scenario, and the participants could indicate their answer on a 5-point scale [1 = ‘not very severe’, 2 = ‘a little severe’, 3 = ‘neither severe or not severe’, 4 = ‘fairly severe’, and 5 = ‘very severe’]. The second question addressed their response as a cyber bystander asking how likely they were to perform the following: (1) Ignore what was happening [passive bystander reaction]. (2) Encourage the pupil that had sent the insulting comment/embarrassing photo or video [joining the bully]. (3) Seek help from a teacher/parent/guardian or trusted adult. (4) Seek help from a friend [seeking help]. (5) Provide emotional support for the pupil that had received the insulting comment/embarrassing photo or video [constructive, victim-focused bystander reaction]. (6) Directly intervene and challenge the pupil [constructive, bully-focused bystander reaction]. Participants could also answer on a 5-point scale [1 = ‘extremely likely’, 2 = ‘somewhat likely’, 3 = ‘neither likely nor unlikely’, 4 = ‘somewhat unlikely’, and 5 = ‘extremely unlikely’]. For the reliability of the subscales see Table 1.

The Empathy Questionnaire for Children and Adolescents (EmQue-CA, [66]) was used to measure empathy. The questionnaire comprises three subscales that measure affective empathy, cognitive empathy, and intention to comfort with a total of 14 items. Respondents were asked to answer on a 3-point scale whether the items were true for them [1 = ‘not true’, 2 = ‘somewhat true’, and 3 = ‘true’]. For the reliability of the subscales see Table 1.

The Cyber Bullying Moral Disengagement Scale (CBMDS, [67]) was used to measure moral disengagement. The 8-item scale measures each moral disengagement mechanism [moral justification, euphemistic language, advantageous comparison, displacement of responsibility, diffusion of responsibility, distorting consequences, attribution of blame, and dehumanising] with one item phrased to refer to cyberbullying situations. Respondents can indicate their answers on a 4-point Likert scale [from 1= ‘don’t agree’ to 4 = ‘totally agree’]. For the reliability of the scale see Table 1.

The Social Desirability Scale (SDS-17, [68]) was used to measure social desirability. The scale comprises 17 socially desirable and undesirable behaviours and respondents can indicate whether the items are describing them or not on a dichotomous scale [0 = ‘false’, 1 = ‘true’]. For the reliability of the scale see Table 1.

Engagement in cyberbullying [cyberbullying perpetration and cybervictimisation] was assessed by using the one-item measure of the Health Behaviour of School-Aged Children study [69,70]. After reading a brief description on what cyberbullying means [e.g., sending/receiving mean messages via text, e-mail or chat, posting mean messages on someone else’s/your social media profile, creating a mocking webpage about someone/you, posting or sending/receiving inappropriate/unflattering photos about someone/you without the victim’s/your consent], respondents were asked to indicate how often in the past couple of months have they perpetrated or experienced the above described scenarios. Respondents had the following answer options: (1) I have not cyberbullied others in the past couple of months./I was not cyberbullied in the past couple of months. (2) Happened once or twice. (3) Happened two or three times in a month. (4) Weekly. (5) Several times a week. For this study an additional question was added to address cyber bystander experiences as well in which the description and question was phrased to be about witnessing cyberbullying. The answer options were phrased the same as for the engagement in cyberbullying questions.

### 2.3. Procedure

To conduct the study, ethical approval was granted from the Research Ethics Committee of Eötvös Loránd University Faculty of Education and Psychology (ref. no.: 2022-633-2). Due to convenience sampling, accessible schools’ school principals were asked whether they would agree to participate in the study. After the approval from the school principals, parents’ informed consent was asked. Before participating in the study, the students were also informed about the nature of the research and asked whether they consented to participate in the study. Two of the students withdrew from the study after reading the information. The study was conducted during school hours in the presence of a research assistant. The questionnaire battery was online, and the students could use their phones or the school’s computers to participate in the study that lasted 45 min. In the end, a debrief form was included that included hotlines and other opportunities (e.g., school psychologist, trusted adult) for the students to ask for help if they struggle with their mental health or have issues related to the topic of the research. Also, before submitting their answers, the students were informed that they could still withdraw from the study if they did not want their answers to be used in the study.

### 2.4. Statistical Analyses

To analyse the data, the IBM SPSS Statistics 25 was used. The first aim of the study was to investigate the perceived severity of the different cyberbullying incidents and to explore how the perceived severity influenced cyber bystander responses. Thus, to analyse the difference between the perceived severity of the three levels of publicity [public, semi-public, private], within-subjects ANOVA was used. To analyse the difference between the perceived severity of the two levels of anonymity [anonymous, not anonymous], type of cyberbullying [visual, written verbal], and victim response [upset, not upset], paired samples *t* tests were used. To explore how the perceived severity levels of all the manipulated variables influenced cyber bystander responses (ignoring, reinforcement of the perpetrator, asking for help from an adult or a friend, emotional support of the victim, direct intervention toward the perpetrator), multiple linear regression analyses were used, where the bystander responses were the dependent variables and the perceived severity of the levels of publicity, anonymity, type of cyberbullying, and victim response were the independent variables.

The second aim of the study was to explore which cyber bystander response was the most likely compared to the others, first regardless of the cyberbullying context and then depending on it. To explore which bystander response [ignorance, reinforcement of the perpetrator, asking for help from an adult or a friend, emotional support of the victim, direct intervention toward the perpetrator] was the most likely compared to the others regardless of the cyberbullying context, we used within-subjects ANOVA with Bonferroni Post Hoc tests to compare the five bystander responses’ likeliness scores. To investigate which cyber bystander response was the most likely in the different manipulated conditions [public, semi-public, private, anonymous, not anonymous, visual, written verbal, victim is upset, and victim is not upset] within-subjects ANOVAs with Bonferroni Post Hoc tests were also used, where in all nine manipulated conditions the five bystander responses’ likeliness scores were compared.

The third aim of the study was to explore the joint effects of contextual and individual variables on the bystander responses. To test not only the effect of the manipulated contextual variables [publicity (public, semi-public, private), anonymity (anonymous, not anonymous), type (written verbal, visual), and victim response (upset, not upset)], but also the individual variables’ [affective empathy, cognitive empathy, intention to comfort, moral disengagement, social desirability, cyberbullying perpetration, and cybervictimisation] effects on the different bystander responses [ignoring, encouraging the cyberbully, asking for help from adult/friend, providing emotional support for the victim, and intervening directly toward the cyberbullying], analyses of covariance (ANCOVAs) were used. In these analyses, the scores of the different responses in the different contexts (e.g., all the ignorance scores in all nine contextual settings) were compared, whereas the individual variables were the covariant variables. To investigate how the significant individual variables are associated with the bystander responses, Pearson correlations were used.

## 3. Results

### 3.1. Perceived Severity of the Cyberbullying Scenarios and Its Effect on Bystander Responses

The first aim of the study was to investigate the cyberbullying incidents’ perceived severity. First, the differences in the perceived severity of the different cyberbullying incidents were analysed depending on the three levels of publicity [using within-subjects ANOVA] and the two levels of anonymity, type of cyberbullying, and the victim’s response [using paired samples *t* tests]. Youngsters perceived that the most severe cyberbullying is when it is public and visual, and when the victim is upset. On the other hand, they perceived private, written verbal cyberbullying when the victim is not upset the least serious scenario. For the descriptive data of all the scenarios and manipulated variables see Table 2.

There were several significant differences in the manipulated instances of the cyberbullying scenarios. According to the results of the within-subjects ANOVA, there was a significant difference between the perceived severity of the levels of publicity [public, semi-public, and private, *F*(1.86, 567.03) = 230.70, *p* < 0.001, *η_p_*^2^ = 0.43]. Indeed, public cyberbullying scenarios [*M* = 23.65, *SD* = 0.41] were deemed to be significantly more serious than semi-public [*M* = 20.86, *SD* = 0.38] and private [*M* = 19.09, *SD* = 0.41]. Furthermore, semi-public cyberbullying scenarios [*M* = 20.86, *SD* = 0.38] were deemed significantly more serious than private ones [*M* = 19.09, *SD* = 0.41]. According to the results of the paired samples *t* tests, visual cyberbullying [*M* = 33.58, *SD* = 10.55] was significantly more severe than written verbal cyberbullying [*M* = 30.02, *SD* = 10.61] and the cyberbullying was significantly more severe when the victim was upset [*M* = 38.23, *SD* = 11.93] than when the victim was not upset [*M* = 25.36, *SD* = 10.25]. There was no significant difference between the anonymous and not anonymous scenarios’ perceived severity. For the detailed results of the paired samples *t* test see Table 3.

Second, the effect of the different types of cyberbullying incidents’ perceived severity on bystander behaviour was analysed using multiple linear regression analyses. The dependent variables were the different bystander responses and the independent variables were the perceived severity scores of the different types of cyberbullying incidents. According to the results, the perceived severity of the different manipulated instances of cyberbullying incidents had a significant effect on all bystander response types: ignorance [*F*(6, 296) = 11.88, *p* < 0.001, *R*^2^ = 0.19], reinforcement of the perpetrator [*F*(6, 293) = 3.06, *p* = 0.01, *R*^2^ = 0.06], asking for help from an adult [*F*(6, 292) = 29.74, *p* < 0.001, *R*^2^ = 0.38] and a friend [*F*(6, 291) = 25.54, *p* < 0.001, *R*^2^ = 0.35], emotional support to the victim [*F*(6, 298) = 28.12, *p* < 0.001, *R*^2^ = 0.36], and intervening by directly challenging the perpetrator [*F*(6, 296) = 14.09, *p* < 0.001, *R*^2^ = 0.22]. From all the variables, only the perceived severity of the visual cyberbullying had a significant effect on the passive bystander response [ignorance, Beta = 0.45, *t* = 2.73, *p* = 0.01]. The perceived severity of public cyberbullying [Beta = −0.41, *t* = −2.09, *p* = 0.04], visual cyberbullying [Beta = −0.36, *t* = −1.97, *p* = 0.05], and the perceived severity of the cyberbullying incident when the victim was not upset [Beta = 0.74, *t* = 2.92, *p* = 0.004] influenced the reinforcement of the perpetrator cyber bystander response. The perceived severity of the written verbal cyberbullying [Beta = 0.39, *t* = 2.63, *p* = 0.01] and the perceived severity when the bystander was not upset [Beta = 0.50, *t* = 2.72, *p* = 0.01] had a significant influence on the asking for help from an adult bystander response, whereas the perceived severity of the public cyberbullying had a marginally significant effect [Beta = −0.27, *t* = −1.73, *p* = 0.08]. Meanwhile, the effect of perceived severity when the victim was upset [Beta = 0.64, *t* = 2.55, *p* = 0.01] and also when the victim was not upset [Beta = 0.70, *t* = 3.29, *p* = 0.001] influenced the bystanders’ asking for help from a friend response. The perceived severity of the visual cyberbullying [Beta = −0.40, *t* = −2.74, *p* = 0.01] and the perceived severity when the victim was both upset [Beta = 0.89, *t* = 3.66, *p* < 0.001] and not upset [Beta = 0.53, *t* = 2.55, *p* = 0.01] had a significant influence on the emotional support bystander response. At last, the perceived severity of not anonymous [Beta = −0.53, *t* = −2.18, *p* = 0.03] and visual [Beta = −0.33, *t* = −2.03, *p* = 0.04] cyberbullying, and the perceived severity when the victim was upset [Beta = 1.12, *t* = 4.11, *p* < 0.001] and also when they were not upset [Beta = 0.88, *t* = 3.83, *p* < 0.001] had significant effect on the direct intervention of bystanders. Further, the perceived severity of public cyberbullying had a marginally significant effect [Beta = −0.32, *t* = −1.82, *p* = 0.07] on this last bystander response.

### 3.2. The Likeliness of the Different Bystander Responses Regardless of the Cyberbullying Context and Depending on It

The second aim of the study was to explore which bystander response is the most likely compared to the others, first, regardless of the effect of the context, then depending on the context. According to the results of the within-subjects ANOVA [*F*(2.22, 639.45) = 147.99, *p* < 0.001, *η_p_*^2^ = 0.34], the most two most likely bystander reactions regardless of the cyberbullying context were ignoring the situation and emotional support to the victim compared to every other cyber bystander responses. The third most likely bystander response was asking help from a friend, which was found to be even more frequent than asking for help from an adult. The second to last bystander response was standing up against the cyberbully (intervene) and the frequency of this was not significantly different from the asking help from an adult bystander response. The least likely bystander response was encouraging the cyberbully compared to every other bystander response type. For the means, standard deviations, and all the other significant Bonferroni Post Hoc tests, see Table 4.

Regarding the results about which cyber bystander response was the most likely in the different contexts, in the cases of the public [*F*(2.33, 708.72) = 133.77, *p* < 0.001, *η_p_*^2^ = 0.31], semi-public [*F*(2.33, 712.27) = 154.24, *p* < 0.001, *η_p_*^2^ = 0.34] and private [*F*(2.19, 662.22) = 154.39, *p* < 0.001, *η_p_*^2^ = 0.62] cyberbullying incidents, the ignoring and the emotional support to the victim bystander responses were the most frequent, and the encouragement of the cyberbully was the least frequent compared to the other bystander responses. Similarly to the different levels of publicity, in the cases of the anonymous [*F*(2.23, 666.74) = 141.33, *p* < 0.001, *η_p_*^2^ = 0.32] and not anonymous [*F*(2.28, 680.50) = 154.50, *p* < 0.001, *η_p_*^2^ = 0.34] cyberbullying incidents, ignoring and emotionally supporting the victim were the two most frequent bystander responses, whereas encouraging the cyberbully was the least frequent. The results were the same for the visual [*F*(2.31, 686.29) = 137.47, *p* < 0.001, *η_p_*^2^ = 0.32] and written verbal [*F*(2.20, 663.30) = 158.86, *p* < 0.001, *η_p_*^2^ = 0.34] cyberbullying contexts, i.e., the ignoring and emotional support bystander responses were the most likely compared to the others and the encouragement of the cyberbullying the least likely response. When the victim was upset, the most likely reaction was the emotional support for the victim and ignoring, respectively, and the least likely was the encouragement of the cyberbully [*F*(2.25, 671.23) = 28.48, *p* < 0.001, *η_p_*^2^ = 0.30]. When the victim was not upset, the most likely bystander response was to ignore the incident and the least likely was to encourage the cyberbully [*F*(2.14, 638.63) = 184.33, *p* < 0.001, *η_p_*^2^ = 0.38]. For the means, standard deviations, and all the other significant Bonferroni Post Hoc tests, see Table 3.

### 3.3. The Joint Effect of Contextual and Individual Variables on Bystander Reactions

The third aim of the study was to investigate the joint effect of the contextual and individual factors on cyber bystander responses. According to the results of the analyses of covariance (ANCOVAs), in the case of the passive bystander response [ignoring], there was a significant difference among the different contexts [*F*(8, 2112) = 18.62, *p* < 0.001, *η_p_*^2^ = 0.07], but also moral disengagement [*F*(8, 2112) = 3.81, *p* < 0.001, *η_p_*^2^ = 0.01], social desirability [*F*(8, 2112) = 3.16, *p* = 0.001, *η_p_*^2^ = 0.01], and affective empathy [*F*(8, 2112) = 3.68, *p* < 0.001, *η_p_*^2^ = 0.01] showed a significant effect on the variance of the ignoring bystander response. Moral disengagement had a positive correlation with the ignoring bystander response [*r*(302) = 0.24, *p* < 0.001], while social desirability [*r*(293) = −0.18, *p* < 0.05] and affective empathy [*r*(307) = −0.26, *p* < 0.001] had a negative correlation with it.

The different contexts’ effect on the variance of the encouraging the cyberbully bystander response was still significant [*F*(8, 2088) = 4.80, *p* < 0.001, *η_p_*^2^ = 0.02], as were the effects of moral disengagement [*F*(8, 2088) = 3.12, *p* < 0.05, *η_p_*^2^ = 0.01] and cyberbullying perpetration [*F*(8, 2088) = 2.02, *p* < 0.05, *η_p_*^2^ = 0.01] as well. The encouraging the cyberbully bystander response had a positive significant correlation with both moral disengagement [*r*(298) = 0.24, *p* < 0.001] and cyberbullying perpetration [*r*(302) = 0.18, *p* = −0.001].

The different contexts’ effect was also significant [*F*(2, 2080) = 2.87, *p* < 0.01, *η_p_*^2^ = 0.01] on the asking help from an adult bystander response’s variance. Furthermore, moral disengagement [*F*(8, 2080) = 4.84, *p* < 0.001, *η_p_*^2^ = 0.02], affective empathy [*F*(8, 2080) = 2.63, *p* = 0.01, *η_p_*^2^ = 0.01], and intention to comfort [*F*(8, 2080) = 2.22, *p* = 0.02, *η_p_*^2^ = 0.01] also had a positive effect on this bystander response’s variance. In addition, social desirability’s effect was marginally significant [*F*(8, 2080) = 1.93, *p* = 0.052, *η_p_*^2^ = 0.01]. Moral disengagement was negatively correlated with the asking help from an adult bystander response [*r*(298) = −0.23, *p* < 0.001], whereas affective empathy [*r*(303) = 0.23, *p* < 0.001], intention to comfort [*r*(297) = 0.22, *p* < 0.001], and social desirability [*r*(289) = 0.21, *p* < 0.001] had a positive correlation with this bystander response.

The contextual difference’s effect was also significant on the asking help from a friend bystander response’s variance [*F*(8, 2064) = 2.37, *p* = 0.02, *η_p_*^2^ = 0.01], and the effects of moral disengagement [*F*(8, 2064) = 2.48, *p* = 0.01, *η_p_*^2^ = 0.01], affective empathy [*F*(8, 2064) = 2.82, *p* < 0.01, *η_p_*^2^ = 0.01], and intention to comfort were also significant [*F*(8, 2064) = 2.55, *p* = 0.01, *η_p_*^2^ = 0.01]. In addition, social desirability had a marginally significant effect [*F*(8, 2064) = 1.72, *p* = 0.09, *η_p_*^2^ = 0.01]. Moral disengagement was negatively correlated with the asking help from a friend bystander response [*r*(295) = −0.23, *p* < 0.001], whereas the correlations with affective empathy [*r*(299) = 0.26, *p* < 0.001], intention to comfort [*r*(293) = 0.25, *p* < 0.001], and social desirability [*r*(286) = 0.18, *p* < 0.01] were positive.

The manipulated contextual factors were not significantly influencing the emotional support for the victim bystander response’s variance [*F*(8, 2120) = 1.52, *p* = 0.15, *η_p_*^2^ = 0.01]. However, moral disengagement [*F*(8, 2120) = 6.47, *p* < 0.001, *η_p_*^2^ = 0.02], social desirability [*F*(8, 2120) = 1.97, *p* = 0.047, *η_p_*^2^ = 0.01], affective empathy [*F*(8, 2120) = 4.05, *p* < 0.001, *η_p_*^2^ = 0.02], cognitive empathy [*F*(8, 2120) = 1.95, *p* = 0.05, *η_p_*^2^ = 0.01], and intention to comfort [*F*(8, 2120) = 2.59, *p* = 0.01, *η_p_*^2^ = 0.01] influenced this bystander response’s variance significantly. The correlation between emotional support for the victim bystander response and moral disengagement was negative [*r*(304) = −0.34, *p* < 0.001], whereas the correlations with affective empathy [*r*(309) = 0.37, *p* < 0.001], cognitive empathy [*r*(305) = 0.25, *p* < 0.001], intention to comfort [*r*(302) = 0.41, *p* < 0.001], and social desirability [*r*(295) = 0.25, *p* < 0.001] were positive.

At last, the contextual difference’s effect on the direct intervention bystander response’s variance was significant [*F*(8, 2120) = 2.15, *p* = 0.03, *η_p_*^2^ = 0.01]. Furthermore, the effects of social desirability [*F*(8, 2120) = 2.18, *p* = 0.03, *η_p_*^2^ = 0.01] and cybervictimisation [*F*(8, 2120) = 1.99, *p* < 0.001, *η_p_*^2^ = 0.01] were significant as well, whereas the effects of moral disengagement [*F*(8, 2120) = 1.90, *p* = 0.06, *η_p_*^2^ = 0.01], cognitive empathy [*F*(8, 2120) = 1.84, *p* = 0.07, *η_p_*^2^ = 0.01], and intention to comfort [*F*(8, 2120) = 1.94, *p* = 0.051, *η_p_*^2^ = 0.01] were marginally significant. The direct intervention bystander response had positive correlation with affective empathy [*r*(307) = 0.16, *p* = 0.01], cognitive empathy [*r*(304) = 0.15, *p* = 0.01], and intention to comfort [*r*(301) = 0.22, *p* < 0.001].

For all the non-significant ANCOVA results see Supplementary Table S2; for all the correlational results see Supplementary Table S3.

## 4. Discussion

The current study aimed to investigate the effect of contextual and individual factors on cyber bystander responses and the results showed both distinct and mutual effects of contextual and individual factors.

The first study aim was to explore the perceived severity of cyberbullying events and its effect on cyber bystander responses. The findings only partially supported our first hypothesis showing that students perceived public and visual cyberbullying when the victim was upset by it the most severe incidents. These results are in line with previous findings also showing that public and visual cyberbullying incidents when the victim was upset were perceived more severely than private and written verbal cyberbullying when the victim was not upset [35,38,39]. However, the present results showed no significant difference in the perceived severity of anonymous and not anonymous cyberbullying incidents. This result is contradictory with previous findings showing that youngsters perceived anonymous cyberbullying more severely than not anonymous incidents [35,38,39]. Our finding is one among many other conflicting results about the role and perception of anonymity in cyberbullying. Some studies showed that youngsters reported that anonymous cyberbullying caused more severe harm on the victim, i.e., feelings of fear, powerlessness, and insecurity [8,48], whereas Nocentini and colleagues [71] have found evidence that being cyberbullied by someone the youngsters know and trust is perceived as more harmful than anonymous cyberbullying acts. The reason of our result could be that anonymity might not be a frequent characteristic of cyberbullying in Hungary, although there is no research evidence supporting this so far, so this is a gap in Hungarian cyberbullying research that needs to be addressed. Furthermore, these contradictory results further support the question of anonymity’s relevance in cyberbullying that also requires further investigation to better fit the theoretical framework of the actual research results.

The second hypothesis proposed that the cyberbullying events that are perceived as highly severe, i.e., public and visual cyberbullying incidents when the victim is upset, will elevate constructive cyber bystander responses. Indeed, our results showed that the perceived severity of these contextually different cyberbullying situations influenced cyber bystander responses, but differently than assumed in the hypothesis, so our preconceptions were not supported. The perceived severity of public cyberbullying incidents decreased the likelihood of reinforcement of the bully, and also of asking for help from an adult and of direct intervention. The publicity of a cyberbullying event might decrease the likelihood of reinforcing the bully because aggressive acts are seen as amoral [56] and youngsters strive to create a positive online image [72]. Furthermore, the publicity of an incident also evokes the ‘bystander effect’ [41,42] and intervening in a public situation might have consequences, e.g., their own subsequent victimisation, thus it is a hindering factor in cyber bystander intervention [29,62]. The reason why adolescents might not be prone to ask for help from an adult might be that adults have different experiences with cyberbullying and different perceptions regarding the consequences and severity of cyberbullying [73,74], or they do not trust that they might be able to provide adequate help or fear judgement or punishment [19,20]. Although youngsters deemed visual cyberbullying (through photos and videos) to be more severe than written cyberbullying, it increased the likelihood of passive cyber bystander responses. Further, visual cyberbullying also decreased the likelihood of reinforcement of the cyberbully, emotional support for the victim, and direct intervention. A probable reason for these results might be that adolescents might not be aware of how they can intervene in such events [33], or they might be so stressed and confused about such events that they ‘freeze’ and are not able to act [31,32]. Another possible reason might be that visual types of cyberbullying might evoke victim blaming and that might hinder constructive cyber bystander responses [26,30]. At last, the severity of cyberbullying situations when the victim was upset increased the likelihood of constructive cyber bystander responses, such as asking for help from a friend, emotional support for the victim, and direct intervention. Previous results showed that hidden reactions from victims hinders cyber bystander intervention [26,30] and our results also support that the victim’s response is a key factor in cyber bystanders’ responses and it is crucial to express their distress following cyberbullying incidents to eventuate constructive cyber bystander responses.

The second study aim was to explore which cyber bystander response was the most likely answer by youngsters and to investigate how the manipulated contextual factors influenced cyber bystander responses. The results showed that the two most likely answers to cyberbullying situations by youngsters were ignoring the situation and emotionally supporting the victim. Similarly, in other studies these two cyber bystander responses were found to be the most frequent ones, although the frequency of them differs. For example, Freis and Gurung [75] found that 76% of the cyber bystanders in their study chose to comfort the victim in some way, and 44% of the sample ‘passed’, yet in another study [76] the majority of the respondents remained passive and fewer than half of the participants showed supportive intervention.

The third hypothesis proposed that constructive cyber bystander responses will be more likely to occur in the event of public anonymous and visual cyberbullying and when the victim is upset, and that non-constructive cyber bystander responses will be more likely to occur in the event of private, not anonymous, and written verbal cyberbullying, when the victim is not upset. According to our results, passive cyber bystander responses (a non-constructive cyber bystander response) and emotional support for the victim (a constructive cyber bystander response) were the most likely in public, semi-public, private, anonymous, not anonymous, and visual and written verbal cyberbullying. So, our hypothesis was only partially supported by our results. This result further supports that youngsters have a limited array of cyber bystander response options. On the other hand, it is a promising result that emotional support for the victim was the other most frequent cyber bystander response.

However, the victims’ reaction context conditions elevated different cyber bystander responses. In the case of an upset victim, the most likely cyber bystander response was to emotionally support the victim (a constructive cyber bystander response), whereas in the case of a not upset victim, the most likely cyber bystander response was to reinforce the bully (a non-constructive and aggressive cyber bystander response). Probably, when the victim does not show any sign of distress, that might mean for the bystanders that indeed this is ‘joking’, so they might feel enabled to join in the ‘fun’. This result might further support that the victim’s reaction might be a prominent contextual factor influencing bystanders.

At last, the research aimed to explore how the individual variables together with the contextual influence cyber bystander responses. The fourth hypothesis proposed that higher levels of empathy, social desirability, and previous cybervictimisation were associated with constructive cyber bystander responses in public, anonymous, and visual cyberbullying when the victim was upset, and it was only partially supported. Affective empathy, intention to comfort, and social desirability were associated with ‘asking for help from an adult and friend’ cyber bystander responses. Direct intervention was associated with social desirability and previous cybervictimisation. In these cases, all the contextual factors also played a role. One of the most interesting results of our study was that in the case of the ‘emotional support of the victim’ cyber bystander response, the contextual variables’ effect disappeared and only the individual variables were shown to play a role: affective and cognitive empathy, social desirability, and intention to comfort had a positive association with this cyber bystander response. Empathy was already found to be associated with prosocial and constructive cyber bystander responses [29,32,41]. Further, these results add to this knowledge by showing that feeling others’ distress vicariously and the intention to comfort others enhance the chance of a constructive bystander response, and emotional support cognitive empathy is also important, i.e., knowing someone’s mental state and recognising their needs while not being overwhelmed by the vicarious feelings [66]. The results also support that adolescents’ cyber bystander responses are motivated by the notion to behave in socially acceptable ways. This might be because for adolescents, it is important to be accepted by their peers and to behave according to peer norms and thus receive positive feedback from them [33,77].

Also, in the fourth hypothesis we presumed that moral disengagement and previous cyberbullying perpetration were associated with cyber bystander responses such as ignorance and reinforcing the perpetrator in private, not anonymous, and written verbal cyberbullying when the victim was not upset. According to our results, moral disengagement was associated with both ignoring and reinforcement of the cyberbully, whereas previous cyberbullying perpetration was only associated with reinforcement of the cyberbully while moral disengagement also had a negative association with all the constructive cyber bystander responses. Indeed, in previous studies [18,24,28,30,31,32] moral disengagement was shown to be associated with passive cyber bystander responses as these strategies justify cyberbullying actions and the lack of involvement as a bystander. Further, a previous study [64] also showed that those who cyberbullied others are more likely to act aggressively as bystanders probably due to the underlying individual, social, and cultural factors such as positive attitude towards cyberbullying, lack of empathy, social support from friends and family, digital parenting and monitoring, cyberbullying legislation, and nationwide digital literacy [78,79,80,81,82].

### Limitations

Our study was not without limitations. Due to the convenience sampling method, the participants of our study are not representative of the Hungarian adolescent population, thus our results cannot be generalised. The cross-sectional correlational design does not allow us to consider causal relations among our variables, so in the future further research is needed. There are several limitations of the vignettes used to measure cyber bystander responses. Adolescents might respond differently to real life cyberbullying situations than they do to vignettes created for research reasons, as the vignettes use verbal descriptions compared to the real-life situation where there are additional visuals, and this limits ecological validity [35]. Further, it is debatable whether vignettes are a reliable and valid research method to capture the fluid nature of cyber bystander responses. Both the perceived severity and bystander response might depend on other variables such as the number of perpetrators, the cyber bystander’s relationship with the perpetrator and/or victim, and action of other cyber bystanders, etc., that were not involved in the vignettes we have used [33] and not controlled for during the analyses. Moreover, the cyber bystander responses were also limited as there are several other options, e.g., bully- or victim-oriented, private or public intervention, and aggression toward the perpetrator [17]. Additionally, adolescents probably reported higher levels of prosocial and constructive bystander responses due to their social desirability (and the results also showed it to be a significant factor). According to the respondents’ feedback to the research assistants, the 24 vignettes were very long and repetitive, which could also possibly influence their motivation to answer honestly and thoroughly. A measurement-related limitation of the study was the low reliability of the affective empathy subscale (Cronbach’s α = 0.64), thus the affective empathy-related results should be interpreted cautiously. The statistical analyses also carry some limitations. We used ANOVAs and ANCOVAs and both of these analyses can lead to false significant results, they are not sufficient to investigate non-linear relationships, they use the assumptions that the data are normally distributed, the variances are homogenous and this impacts validity, and they are sensitive to measurement errors in the covariates, hence they can reduce statistical power. Thus, our results should be interpreted carefully.

## 5. Conclusions

To answer our research question, i.e., ‘to ignore, to join in, or to intervene?’, our research showed that in all cyberbullying contexts, the passive cyber bystander response (ignorance) and the emotional support for the victim (a form of intervention) were the two most likely cyber bystander responses, and reinforcement of the cyberbully (i.e., joining in) was in all contexts the least likely. Furthermore, our research also showed the specific contextual and individual variables that were associated with the different cyber bystander responses. The passive cyber bystander response was associated with the perceived severity of visual cyberbullying, high moral disengagement, low social desirability, and low affective empathy. The emotional support for the victim was the only cyber bystander response that was associated with only the individual variables, i.e., low moral disengagement, high social desirability, high affective and cognitive empathy, and high intention to comfort. The perceived severity of written verbal cyberbullying and when the victim was not upset, low moral disengagement, high affective empathy, and high intention to comfort were associated with the asking for help from an adult cyber bystander response. The perceived severity of the victim upset and not upset cyberbullying scenarios, low moral disengagement, high affective empathy, and high intention to comfort were associated with the asking for help from a friend cyber bystander response. Direct intervention in cyberbullying incidents was associated with the perceived severity of not anonymous and visual cyberbullying, when the victim was and was not upset, and high empathic skills. The encouragement of the cyberbully was associated with the perceived severity of public and visual cyberbullying, and when the victim was not upset, high moral disengagement, and previous cyberbullying perpetration. To conclude, the role of the perceived severity of the cyberbullying contexts varied among the cyber bystander responses, while the results showed the prominent role of all cyberbullying contexts, moral disengagement, empathy, and as a new variable, social desirability in cyber bystander responses.

The severity and consequences of anonymous cyberbullying acts need further research due to the inconsistent results regarding their role both in cyberbullying itself and on cyber bystanders. Our results also further support the importance of prevention and the elements that would be crucial to be included in prevention programmes when targeting passive bystanders [15]. It would be cardinal to teach strategies to adolescents on how they can intervene in different ways and show how all the different strategies could be viable options for them as previous studies [33] showed they lack this knowledge and in our research they also chose a limited array of the possible cyber bystander responses. Moreover, prevention should also emphasise the importance of how someone can show their distress online and ask for help if they are cybervictimised, and future research should also concentrate on investigating the effects of victims’ distress signals. At last, focusing on enhancing socio-emotional skills, specifically empathy training and decreasing the use of moral disengagement strategies, should also be significant parts of cyberbullying prevention.

## Figures and Tables

**Table 1:** Reliability of the subscales.

Subscales	Cronbach’s Alpha
Perceived severity of cyberbullying incidents	0.95
Ignorance	0.97
Reinforcing the cyberbullying perpetrator	0.98
Seeking help from an adult	0.98
Seeking help from a friend	0.98
Emotional support for the victim	0.98
Direct intervention	0.98
Affective empathy	0.64
Cognitive empathy	0.76
Intention to comfort	0.76
Moral disengagement	0.80
Social desirability	0.69

**Table 2:** The mean and standard deviation (SD) of perceived severity across scenarios.

Scenario	Publicity	Anonymity	Type of Cyberbullying	Victim Response	Perceived Severity
	Semi-Public	Not Anonymous	Visual	Not Upset	2.35 (1.12)
	Semi-Public	Anonymous	Written Verbal	Upset	2.93 (1.18)
	Public	Anonymous	Visual	Upset	3.88 (1.14)
	Public	Not Anonymous	Written Verbal	Upset	3.33 (1.23)
	Semi-Public	Not Anonymous	Written Verbal	Not Upset	2.01 (1.10)
	Private	Not Anonymous	Written Verbal	Upset	2.83 (1.27)
	Public	Not Anonymous	Visual	Not Upset	2.63 (1.29)
	Public	Anonymous	Written Verbal	Not Upset	2.30 (1.22)
	Semi-Public	Not Anonymous	Written Verbal	Upset	2.96 (1.29)
	Semi-Public	Not Anonymous	Visual	Upset	3.23 (1.25)
	Private	Not Anonymous	Written Verbal	Not Upset	1.69 (1.07)
	Public	Not Anonymous	Written Verbal	Not Upset	2.16 (1.15)
	Public	Anonymous	Visual	Not Upset	2.48 (1.23)
	Private	Not Anonymous	Visual	Not Upset	1.90 (1.15)
	Private	Anonymous	Visual	Not Upset	2.00 (1.16)
	Private	Anonymous	Written Verbal	Not Upset	1.82 (1.05)
	Public	Not Anonymous	Visual	Upset	3.06 (1.34)
	Private	Not Anonymous	Visual	Upset	3.04 (1.29)
	Semi-Public	Anonymous	Visual	Upset	3.30 (1.28)
	Public	Anonymous	Written Verbal	Upset	3.32 (1.41)
	Semi-Public	Anonymous	Written Verbal	Not Upset	1.94 (1.10)
	Semi-Public	Anonymous	Visual	Not Upset	2.18 (1.17)
	Private	Anonymous	Written Verbal	Upset	2.85 (1.33)
	Private	Anonymous	Visual	Upset	3.08 (1.33)

**Table 3:** Results of the paired samples *t* tests.

Pairs	*t*	*df*	*p*	95% Confidence Interval of the Difference	
				Lower	Upper
Anonymous–Not anonymous	1.76	305	0.08	−0.05	−0.94
Written verbal–Visual	−9.19	305	<0.001	−4.32	−2.79
Victim upset–Victim not upset	23.34	305	<0.001	11.78	13.95

**Table 4:** The mean and standard deviations of the different cyber bystander response types in the different cyberbullying contexts.

Context	Type or Bystander Response						Sig. Bonferroni Post Hoc Test
	Ignore	Encourage	Adult Help	Friend Help	Emotional Support	Intervene	
Total	76.45 (29.51)	30.64 (18.03)	51.48 (27.18)	58.56 (27.39)	68.52 (29.61)	48.84 (24.93)	1-2, 1-3, 1-4, 1-6, 2-3, 2-4, 2-5, 2-6, 3-4, 3-5, 4-5, 4-6, 5-6
Public	24.10 (9.89)	10.24 (5.98)	18.31(9.31)	20.63 (9.43)	23.75 (10.04)	17.41 (8.74)	1-2, 1-3, 1-4, 1-6, 2-3, 2-4, 2-5, 2-6, 3-4, 3-5, 4-5, 4-6, 5-6
Semi-Public	25.29 (9.50)	10.53 (6.10)	17.53 (9.18)	20.28 (9.19)	23.78 (9.89)	16.95 (8.43)	1-2. 1-3, 1-4, 1-6, 2-3, 2-4, 2-5, 2-6, 3-4, 3-5, 4-5, 4-6, 5-6
Private	26.34 (10.74)	10.04 (6.15)	16.24 (9.19)	18.02 (9.46)	21.72 (10.29)	15.63 (8.61)	1-2, 1-3, 1-4, 1-5, 1-6, 2-3, 2-4, 2-5, 2-6, 3-4, 3-5, 4-5, 4-6, 5-6
Anonymous	37.76 (15.21)	15.23 (9.13)	26.06 (13.64)	29.32 (13.89)	34.15 (15.03)	24.84 (12.96)	1-2, 1-3, 1-4, 1-6, 2-3, 2-4, 2-5, 2-6, 3-4, 3-5, 4-5, 4-6, 5-6
Not Anonymous	37.98 (14.42)	15.58 (9.06)	26.03 (13.69)	29.60 (13.82)	35.10 (14.87)	25.15 (12.60)	1-2, 1-3, 1-4, 1-6, 2-3, 2-4, 2-5, 2-6, 3-4, 3-5, 4-5, 4-6, 5-6
Visual	36.81 (14.87)	15.36 (8.96)	26.91 (13.86)	30.23 (14.13)	35.22 (14.86)	25.48 (12.88)	1-2, 1-3, 1-4, 1-6, 2-3, 2-4, 2-5, 2-6, 3-4, 3-5, 4-5, 4-6, 5-6
Written Verbal	38.92 (14.86)	15.45 (9.19)	25.18 (13.60)	28.70 (13.80)	34.03 (15.02)	24.50 (12.67)	1-2, 1-3, 1-4, 1-5, 1-6, 2-3, 2-4, 2-5, 2-6, 3-4, 3-5, 4-5, 4-6, 5-6
Victim Upset	34.27 (15.14)	15.27 (8.85)	28.58 (14.40)	32.73 (14.66)	38.29 (15.52)	27.56 (13.97)	1-2, 1-3, 1-6, 2-3, 2-4, 2-5, 2-6, 3-4, 3-5, 4-5, 4-6, 5-6
Victim Not Upset	41.46 (15.56)	15.55 (9.34)	23.51 (13.59)	26.20 (13.81)	30.96 (15.15)	22.43 (12.30)	1-2, 1-3, 1-4, 1-5, 1-6, 2-3, 2-4, 2-5, 2-6, 3-4, 3-5, 4-5, 4-6, 5-6

1 = Ignore, 2 = Encourage, 3 = Adult help, 4 = Friend help, 5 = Emotional support, 6 = Intervene.

## Data Availability

The original contributions presented in this study are included in the article/Supplementary Material. Further inquiries can be directed to the corresponding author.

## Supplementary Materials

The following supporting information can be downloaded at https://www.mdpi.com/article/10.3390/children13010113/s1. Table S1: Frequency of cyber bystander, cyberbullying perpetrator and cybervictimization experiences; Table S2: Non-significant results of the ANCOVAs; Table S3: Results of the Pearson correlations between the bystander responses and the individual variables.
