# Supplementary material for: To Ignore, to Join in, or to Intervene? Contextual and Individual Factors Influencing Cyber Bystanders’ Response to Cyberbullying Incidents
Source: Children (Basel). 2026 Jan 12;13(1):113. doi: 10.3390/children13010113 (PMC12840138; doi:10.3390/children13010113)
Supplement: Supplementary file 1 [file children-13-00113-s001.zip › children-4037173-supplementary.pdf]

Supplementary Table S1. Frequency of cyber bystander, cyberbullying perpetrator and cybervictimization experiences

Frequency	Cyber bystander	Cyberbullying perpetrator	Cybervictimization
Never	61.1%	76.8%	74.2%
Once or twice	27.4%	13.7%	15.0%
Two or three times a month	4.8%	1.6%	4.1%
Weekly	2.5%	1.3%	1.9%
Several times a week	3.8%	5.7%	3.8%

Supplementary Table S2. Non-significant results of the ANCOVAs

Bystander response*factor	*F(df)*	*p*	η_p^2
Ignore*cognitive empathy	.39(8,2112)	.92	.001
Ignore*intention to comfort	.38(8,2112)	.93	.001
Ignore*cyberbullying perpetration	.47(8,2112)	.88	.002
Ignore*cybervictimization	.53(8,2112)	.83	.002
Encourage*social desirability	.46(8,2088)	.89	.002
Encourage*affective empathy	.49(8,2088)	.86	.002
Encourage*cognitive empathy	.87(8,2088)	.54	.003
Encourage*intention to comfort	1.54(8,2088)	.14	.01
Encourage*cybervictimization	.37(8,2088)	.94	.001
Adult help*cognitive empathy	.63(8,2080)	.75	.002
Adult help*cyberbullying perpetration	.99(8,2080)	.44	.004
Adult help*cybervictimization	.43(8,2080)	.77	.002
Friend help*cognitive empathy	1.53(8,2064)	.14	.01
Friend help*cyberbullying perpetration	.18(8,2064)	.99	.001
Friend help*cybervictimization	.19(8,2064)	.99	.001
Emotional support (only context)	1.52(8,2120)	.15	.01
Emotional support*cyberbullying perpetration	.41(8,2120)	.91	.002
Emotional support*cybervictimization	.78(8,2120)	.62	.003
Intervene*affective empathy	.79(8,2120)	.61	.003
Intervene*cyberbullying perpetration	.45(8,2120)	.89	.002

Supplementary Table S3. Results of the Pearson correlations between the bystander responses and the individual variables.

		Moral disengagement	Social desirability	Affective empathy	Cognitive empathy	Intention to comfort	Cyberbullying perpetration	Cybervictimization
Ignore	*r(df)*	.24(302)**	-.18(293)**	-.26(307)**	-.08(304)	-.17(301)**	.11(306)*	.04(306)
Encourage	*r(df)*	.23(298)**	-07(289)	-.05(303)	-.01(301)	-.11(297)	.18(302)**	.10(302)
Adult help	*r(df)*	-.23(298)**	.21(289)**	.23(303)**	.09(300)	.22(297)**	-.04(302)	-.02(302)

Friend help	*r(df)*	-.23(295)**	.18(286)**	.26(299)**	.09(296)	.25(293)**	-.08(298)	-.02(300)
Emotional support	*r(df)*	-.34(304)**	.25(295)**	.37(309)**	.25(305)**	.41(303)**	-.07(308)	.04(308)
Intervene	*r(df)*	-.08(302)	.06(294)	.16(307)**	.15(304)*	.22(301)**	.06(306)	.11(306)

$^{**}p<.01$; $^{*}p<.05$
